# Metabolomic Signatures of Autism Spectrum Disorder

**DOI:** 10.3390/jpm12101727

**Published:** 2022-10-17

**Authors:** Danielle Brister, Shannon Rose, Leanna Delhey, Marie Tippett, Yan Jin, Haiwei Gu, Richard E. Frye

**Affiliations:** 1 College of Liberal Arts and Sciences, School of Molecular Sciences, Arizona State University, Tempe, AZ 85281, USA; 2 Arkansas Children’s Research Institute and Department of Pediatrics, University of Arkansas for Medical Sciences, Little Rock, AR 72202, USA; 3 Center for Translational Science, Florida International University, Port St. Lucie, FL 34987, USA; 4 Rossignol Medical Center, Phoenix, AZ 85050, USA

**Keywords:** amino acid metabolism, autism spectrum disorder, energy metabolism, mass spectrometry, metabolomics, mitochondria, redox metabolism

## Abstract

Autism Spectrum Disorder (ASD) is associated with many variations in metabolism, but the ex-act correlates of these metabolic disturbances with behavior and development and their links to other core metabolic disruptions are understudied. In this study, large-scale targeted LC-MS/MS metabolomic analysis was conducted on fasting morning plasma samples from 57 children with ASD (29 with neurodevelopmental regression, NDR) and 37 healthy controls of similar age and gender. Linear model determined the metabolic signatures of ASD with and without NDR, measures of behavior and neurodevelopment, as well as markers of oxidative stress, inflammation, redox, methylation, and mitochondrial metabolism. MetaboAnalyst ver 5.0 (the Wishart Research Group at the University of Alberta, Edmonton, Canada) identified the pathways associated with altered metabolic signatures. Differences in histidine and glutathione metabolism as well as aromatic amino acid (AAA) biosynthesis differentiated ASD from controls. NDR was associated with disruption in nicotinamide and energy metabolism. Sleep and neurodevelopment were associated with energy metabolism while neurodevelopment was also associated with purine metabolism and aminoacyl-tRNA biosynthesis. While behavior was as-sociated with some of the same pathways as neurodevelopment, it was also associated with alternations in neurotransmitter metabolism. Alterations in methylation was associated with aminoacyl-tRNA biosynthesis and branched chain amino acid (BCAA) and nicotinamide metabolism. Alterations in glutathione metabolism was associated with changes in glycine, serine and threonine, BCAA and AAA metabolism. Markers of oxidative stress and inflammation were as-sociated with energy metabolism and aminoacyl-tRNA biosynthesis. Alterations in mitochondrial metabolism was associated with alterations in energy metabolism and L-glutamine. Using behavioral and biochemical markers, this study finds convergent disturbances in specific metabolic pathways with ASD, particularly changes in energy, nicotinamide, neurotransmitters, and BCAA, as well as aminoacyl-tRNA biosynthesis.

## 1. Introduction

Autism spectrum disorder (ASD) is one of the most concerning medical problems of our era. ASD continues to increase in prevalence with the most recent Autism and Developmental Disabilities Monitoring Network estimates suggesting that 1 in 44 children have the disorder [1] and a more recent study estimating that it might reach a prevalence of 1 in 30 [2]. Despite studying ASD for decades [3] well-validated objective biomarkers to assist with the diagnosis are still lacking with most biomarkers in the early stages of development [4]. Examination of potential physiological markers associated with ASD reveal that metabolic and immune biomarkers are promising, consistent with converging evidence that suggests that mitochondrial dysfunction and oxidative stress are common factors in many neurodevelopmental disorders [5,6,7,8] and interact with immune system activation to create a pathophysiological disturbance known as the ‘Bad Trio’ [9].

ASD is associated with several systemic metabolic abnormalities. Although only 5% of children with ASD exhibit classically mitochondrial disease [10], up to 80% demonstrate biomarkers of abnormalities in energy metabolism [11]. Abnormal biomarkers of carbohydrate-linked mitochondrial dysfunction such as lactate, pyruvate, and alanine [10] as well as fatty acid oxidation biomarkers [12,13] are prevalent in the ASD population. Although biomarkers of fatty acid metabolism are reported as blood-based biomarkers, such abnormalities have been found in the stool [14] and brain [15] in children with ASD. Abnormalities in folate one-carbon metabolism (FOCM) are so prevalent in ASD that they have recently been proposed to be potentially diagnostic [16,17,18]. Importantly, abnormalities in FOCM have downstream effects on methylation, transsulfuration, redox metabolism, and oxidative stress [19,20].

Studies on blood amino acids in ASD tend to find variable results, but, overall, studies find lower overall amino acid concentrations in the blood [10] and urine [21]. A large prospective, controlled non-randomized multisite study found dysregulated branched chain amino acid (BCAA) metabolism in 17% of children with ASD, as well as dysregulated glutamine, glycine, and ornithine in smaller ASD subgroups [22]. Other studies have implicated tryptophan and tyrosine, two important neurotransmitter precursors, as dysregulated in ASD [23,24,25], but some have suggested that these changes may be related to restricted diets commonly associated with ASD [26]. Consistent with reduced neurotransmitter amino acid precursors, studies have implicated abnormalities in monoamine neurotransmitters themselves [27]. Abnormalities in central folate [28] and tetrahydrobiopterin [29], cofactors required to convert precursors to neurotransmitters, could also explain monoamine neurotransmitter abnormalities. 

One interesting finding in previous studies that might link many of the aforementioned metabolic abnormalities is disruption of niacin metabolism. De novo synthesis of niacin in the human body utilizes the essential amino acid tryptophan [30], and niacin is the precursor for nicotinamide adenine dinucleotide (NAD+) and nicotinamide adenine dinucleotide phosphate (NADP+), which are cofactors essential for may metabolic reactions including redox and energy metabolism [31]. Biomarkers of nicotinamide have been found to be abnormal in the urine [32,33] and stool [14] in children with ASD. 

One complication of metabolic biomarker studies is the large number of metabolites considered in the analysis, making any one metabolite often non-specific. Thus, for such analysis, pathway analysis is often helpful to better understand the complex data [34]. In this study, we use pathway analysis and analysis of metabolic-metabolic interactions to better understand metabolic signatures associated with ASD. Many studies try to discover a set of biomarkers which separate individuals with ASD from TD individuals, but such an approach minimizes that significance of the profound heterogenicity associated with the ASD population. Thus, in this study, the heterogenicity in the ASD sample is used to better understand metabolomic abnormalities. Measures of behavior and cognition as well as specific measures of metabolic derangements associated with ASD are correlated with the metabolomic measurements to better understand the variation in metabolic abnormalities associated with ASD and their significance.

## 2. Materials and Methods

### 2.1. Participants 

The participants in this study represent a subset of previously published cohorts [35,36]. Thus, some of the methods can be found in our previous publications, although they are briefly outlined here. The protocol was approved by the Institutional Review Board at the University of Arkansas for Medical Sciences (Little Rock, AR, USA), and the study is registered in clinicaltrials.gov (NCT02000284). Parents of participants provided written informed consent. Participants characteristics are given in Table S1.

Exclusion criteria were (i) chronic treatment with medications that would detrimentally affect mitochondrial function such as antipsychotic medications; (ii) vitamin or mineral supplementation exceeding the recommended daily allowance, and (iii) prematurity. Inclusion criteria included the ability to tolerate phlebotomy and, for those with ASD, a diagnosis of ASD as documented using our previous published criteria [35,36]. Typically developing (TD) children were include if they did not have any neurological disorders or developmental delays, and ASD symptoms were ruled out with a Social Communication Questionnaire score <12. Children underwent a fasting blood draw in the morning. 

The neurodevelopmental regression (NDR) history was obtained using the Developmental and Neurobehavioral Regression (DANR) questionnaire which is described in detail elsewhere [35]. The DANR records detail information about NDR including specific questions on premorbid functioning before the regression, duration of the regression, specific skills lost and when the skills were regained, whether there was a single or multiple regressions and any known trigger such as illness, fever, or seizure. 

### 2.2. Behavioral Measurements

As standard practice for our laboratory [37,38], research staff were trained by a multispecialty team consisting of two licensed psychologists and a speech therapist prior to performing assessments. During the study, a research psychologist supervised research staff and provided feedback and retraining if necessary. The Vineland Adaptive Behavior Scale (VABS) 2nd edition was completed using the Survey Interview Form, and parents completed the Aberrant Behavior Checklist (ABC) and the Social Responsiveness Scale (SRS). These validated measures provide an assessment of neurodevelopment and ASD symptoms in children with ASD [37,38]. Sleep was assessed with the standard tool for documenting sleep abnormalities in ASD, the Childhood Sleep Habits Questionnaire (CSHQ) [39]. Core Language (CL) was assessed by the most appropriate instrument given the participants age, either the Clinical Evaluation of Language Fundamentals (CELF)-preschool-2, CELF-4, or the Preschool Language Scale-5 (PLS-5). The standardized summary score of each instrument (mean 100, standard deviation 15) was used as the measure of core language. 

### 2.3. Sample Collections and Storage 

Up to 20 mL of blood was collected into an ethylenediaminetetraacetic acid (EDTA)-Vacutainer tube, chilled on ice, and centrifuged at 1500× *g* for 10 min at 4 °C to separate plasma within 30 min of collection. Plasma was removed and stored at −80 °C for later analysis and replaced with room temperature wash buffer containing Ca^+2^/Mg^+2^-free PBS with 0.1% BSA and 2 mM EDTA. Diluted blood was then layered on top of Histopaque-1077 (Sigma Aldrich, St. Louis, MO, USA) and centrifuged at 400× *g* for 30 min at room temperature. Peripheral blood mononuclear cells (PBMCs) were washed twice with wash buffer and counted using a hemocytometer. Viability exceeded 95% in all cases and recovery was approximately 10^6^/mL in most cases. Isolation procedure duration was 90–120 min. Fresh PBMCs were used for the mitochondrial respiration assay.

### 2.4. Redox Biomarkers

High Performance Liquid Chromatography with electrochemical detection was used to measure free and total reduced glutathione (GSH), as well as oxidized glutathione (GSSG) from which the free (fGSH/GSSG) and total (tGSH/GSSG) glutathione redox ratios were calculated. Intracellular redox ratio (iGSH/GSSG) from frozen PBMC was also measured. Methylation potential was measured as the S-adenosylmethionine (SAM) to S-adenosylhomocysteine (SAH) ratio. Finally, 3-nitrotyrosine (3-NT), a measure of oxidative damage to proteins, and 3-chlorotyrosine (3-CT), a measure of immune activity, were also measured. Measurements were performed within 2 weeks of collection [40,41].

### 2.5. Mitochondrial Respiration Assay

Bioenergetic data was obtained from fresh PBMCs the same day as collection using a state-of-the-art Seahorse XFe96 Analyzer (Seahorse Bioscience, Inc., North Billerica, MA, USA). The Seahorse analyzer measures oxygen consumption rate (OCR) in real-time in a 96-well plate in a wide range of intact living cell types [42,43]. The assay measures several key parameters: Adenosine triphosphate (ATP) Linked Respiration (ALR) and Proton Leak Respiration (PLR). Mitochondrial function in PBMCs of children with ASD has been measured using the Seahorse XFe96 in siblings with ASD and genetic abnormalities [44], in children being evaluated for immune abnormalities to demonstrate the correlation between cytokine profiles and mitochondrial function in children with ASD [45] and in a cohort of children with ASD with and without NDR [46]. 

PBMCs were placed in assay media (unbuffered RPMI supplemented with 1 mM pyruvate, 2 mM glutamate, and 25 mM glucose) that was warmed to 37 °C and pH adjusted to 7.4 prior to cell suspension. XFe96 plates (Seahorse Bioscience, Billerica, MA, USA) were prepared by adding 25 μL of 50 μg/mL Poly-D-lysine (EMD Millipore, Billerica, MA, USA) for two hours, washing with 250 μL sterile water and drying in a laminar flow hood overnight prior to seeding with 4 × 10^5^ viable PBMCs per well. After seeding, the plates were spun with slow acceleration (4 on a scale of 9) to a maximum of 100 g for 2 min and then allowed to stop with zero braking (Eppendorf Model 5810R Centrifuge). The plate orientation was reversed, and the plate was spun again to 100 g in the same fashion. Prior to Seahorse assay, XFe96 wells were visualized using an inverted microscope to ensure PBMCs were evenly distributed in a single layer and viability of the cells was confirmed by trypan blue exclusion. Four replicate samples were measured simultaneously to improve assay reliability. Runs with clear measurement probe failure, reagent injection failures, or non-physiology measurements (ALR or PLR < −1 pmol/min) were eliminated. In our previous study, measurement reliability was found to be excellent [35]. 

### 2.6. Metabolomic Analysis

Procedures of metabolomic analysis have been outlined previously [34] but the procedures are outlined here for completeness.

#### 2.6.1. Sample Processing

Frozen samples were first thawed overnight under 4 °C, and 50 μL of each sample was placed in a 2 mL Eppendorf vial. The initial step for protein precipitation and metabolite extraction was performed by adding 500 μL MeOH and 50 μL internal standard solution (containing 1810.5 μM ^13^C_3_-lactate and 142 μM ^13^C_5_-glutamic acid). The mixture was then vortexed for 10s and stored at −20 °C for 30 min, followed by centrifugation at 14,000 RPM for 10 min at 4 °C. The supernatant (450 μL) was collected into a new Eppendorf vial, and dried using a CentriVap Concentrator (Labconco, Fort Scott, KS, USA). The dried samples were reconstituted in 150 μL of 40% PBS/60% ACN.

#### 2.6.2. Reagents 

Acetonitrile (ACN), methanol (MeOH), ammonium acetate, and acetic acid, all LC-MS grade, were purchased from Fisher Scientific (Pittsburgh, PA, USA). Ammonium hydroxide was bought from Sigma-Aldrich (Saint Louis, MO, USA). DI water was provided in-house by a Water Purification System from EMD Millipore (Billerica, MA, USA). PBS was bought from GE Healthcare Life Sciences (Logan, UT, USA). The standard compounds corresponding to the measured metabolites were purchased from Sigma-Aldrich (Saint Louis, MO, USA) and Fisher Scientific (Pittsburgh, PA, USA).

#### 2.6.3. LC-MS/MS Method

The large-scale targeted LC-MS/MS method is widely used [47,48,49,50,51,52]. LC-MS/MS experiments were performed on an Agilent 1290 UPLC-6490 QQQ-MS (Santa Clara, CA, USA) system. Each sample was injected twice, 10 µL for analysis using negative ionization mode and 4 µL for analysis using positive ionization mode. Both chromatographic separations were performed in hydrophilic interaction chromatography (HILIC) mode on a Waters XBridge BEH Amide column (150 × 2.1 mm, 2.5 µm particle size, Waters Corporation, Milford, MA, USA). The flow rate was 0.3 mL/min, auto-sampler temperature was kept at 4 °C, and the column compartment was set at 40 °C. The mobile phase was composed of Solvents A (10 mM ammonium acetate, 10 mM ammonium hydroxide in 95% H2O/5% ACN) and B (10 mM ammonium acetate, 10 mM ammonium hydroxide in 95% ACN/5% H_2_O). After the initial 1 min isocratic elution of 90% B, the percentage of Solvent B decreased to 40% at t = 11 min. The composition of Solvent B maintained at 40% for 4 min (t = 15 min), and then the percentage of B gradually went back to 90%, to prepare for the next injection. 

The mass spectrometer was equipped with an electrospray ionization (ESI) source. Targeted data acquisition was performed in multiple-reaction-monitoring (MRM) mode. The whole LC-MS system was controlled by Agilent Masshunter Workstation software (Santa Clara, CA, USA). The extracted MRM peaks were integrated using Agilent MassHunter Quantitative Data Analysis (Santa Clara, CA, USA). A pooled sample, which was a mixture of all blood samples was used as the quality-control (QC) sample. We ran the QC once every 10 study samples to ensure the good data quality.

### 2.7. Data Analysis

Metabolomics data was reviewed, and compounds with > 20% coefficient of variation or without measurable peaks in >20% of all the samples were eliminated. MetaboAnalyst 5.0 (Wishart Research Group, University of Alberta, Edmonton, Canada) was used for data analysis [53]. Data were scaled using Log_10_ transformation and auto-scaling (mean-centered and divided by the standard deviation of each variable) (See Figure S1).

Linear Model Analysis was used to define the significantly different metabolites from one disease group (ASD) and two sub disease groups (ASD NDR, ASD No NDR) as compared to control (CNT) group. Correlation Analysis was used to define significant metabolites that were correlated to participants from both ASD and CNT groups and their behavioral scores from the CL, VABS, SRS, CSHQ, and ABC. Correlation Analysis was also used to define significant metabolomic measurements with methylation potential, tGSH/GSSG, fGSH/GSSG, iGSH/GSSG, 3-NT and 3-CT. 

Alpha was set to ≤0.05 to identify potential important metabolites at the risk of incorrectly identifying metabolites which are not important. Since the metabolites identified were then entered into pathway analysis, metabolites which may be erroneously identified most likely will be eliminated as they will not be consistent with other metabolites identified in common pathways. Furthermore, by examining the relationships between behavior and metabolic changes, the common pathways which converge across multiple analyses can be identified. Regarding statistical power of the comparisons in this study, the average statistic across comparisons was used to calculate the effect size. For group comparisons an t-value of 2.5 resulted in a medium effect size of a Cohen d’ of 0.52 which provides a power of 68%. For correlation analysis an average r of 0.3 is a medium effect size and provides an 85% power. G * Power v 3.1.9.7 (Universität Kiel, Germany) was used for the power calculations. 

Key metabolites identified by linear models and correlation were investigated using the pathway and metabolite-metabolite interaction tool of the network analysis package MetaboAnalyst 5.0. Metabolite networks were analyzed in detail. The major key metabolites were determined by decreasing node scope to minimum number of nodes to the top-level nodes.

## 3. Results

### 3.1. Linear Models

**Figure 1:**
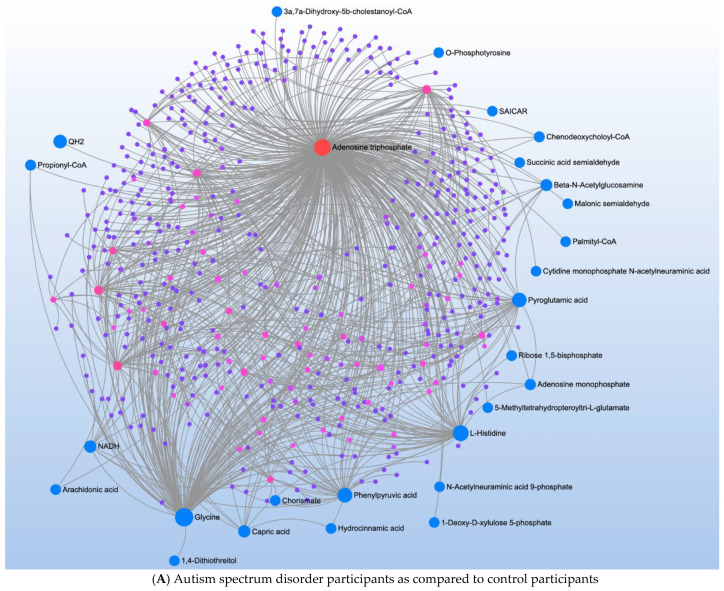

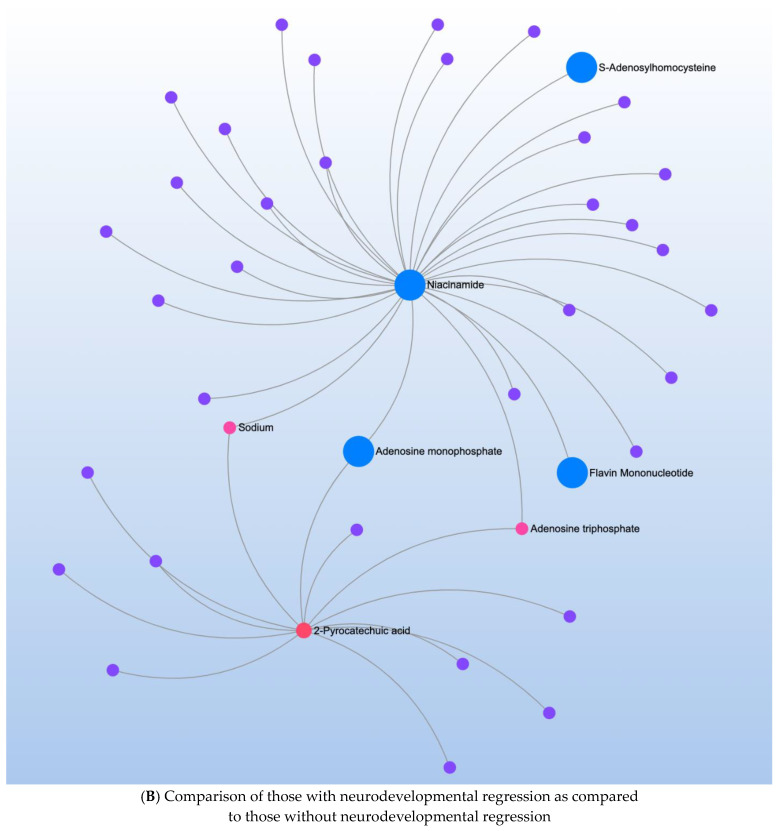
Metabolite-metabolite interaction network for significant metabolites that differentiate participants with (**A**) autism spectrum disorder (ASD) from typically developing controls and (**B**) those with ASD with and without neurodevelopmental regression (NDR). Size of the node indicates importance in the network. The network highlights that importance of adenosine triphosphate in differentiating those with and without ASD and those with ASD but with and without NDR. The importance of nicotinamide metabolism in differentiating those with and without NDR is also highlighted by the interaction analysis.

Comparison of ASD vs. CNT found 23 significant metabolites associated with three significant pathways (Table 1). Metabolite-metabolite interaction analysis confirmed the central role of L-Histidine, Glycine, and Pyroglutamic acid but also demonstrated a central role of ATP (Figure 1A). 

A comparison of ASD individuals with and without NDR identified three significant metabolites which mapped to one significant pathway (Table 2). Metabolite-metabolite analysis confirmed the importance of nicotinamide metabolism and emphasized its link to ATP production (Figure 1B). 

**Figure 2:**
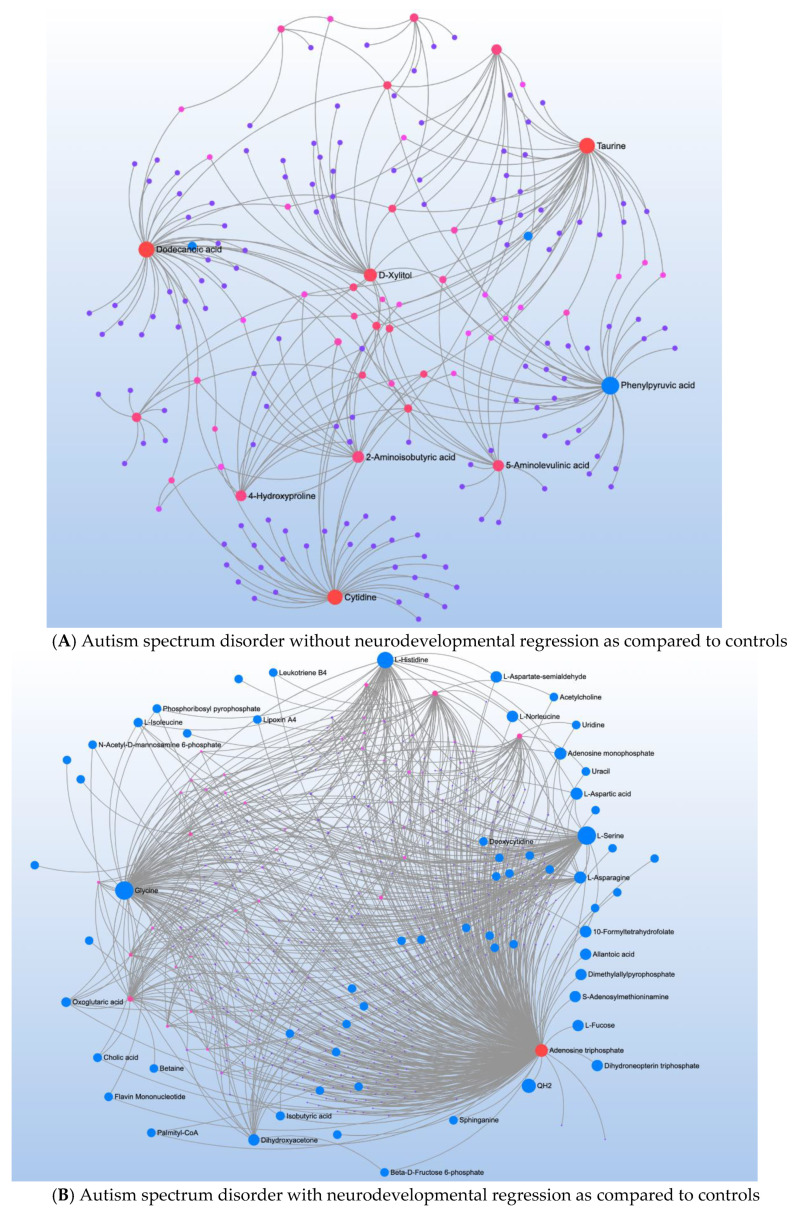
Metabolite-metabolite interaction network for significant metabolites that differentiate participants with (**A**) autism spectrum disorder (ASD) from typically developing controls. Size of the node indicates importance in the network. There were multiple metabolites that appeared to differential the individuals with ASD without a history of neurodevelopmental regression (NDR) from control (**B**), particularly phenylpyruvic acid, a phenylalanine derivative. Controls were differentiated from those with ASD and a history of NDR by several metabolites, particularly adenosine triphosphate.

Comparison of CNT to individuals with ASD but without NDR found 18 significant metabolites which mapped to the aromatic amino acid (AAAs) pathway (Table 3). However, this pathway had low impact, most likely because these metabolites represented many diverse pathways (Figure 2A). Indeed, metabolite-metabolite analysis identified several central metabolites including dodecanoic and capric acid (both medium chain triglycerides), phenylpyruvic acid (a phenylalanine derivative), cytidine (a pyrimidine involved in neuronal-glial glutamate cycling), and taurine (involved in transsulfuration metabolism).

Comparing CNT to individuals with ASD with NDR found 19 significant metabolites which mapped to four significant pathway (Table 4). Metabolite-metabolite analysis converged on ATP which strongly implicates mitochondrial function in NDR (Figure 2B).

### 3.2. Behavioral Correlations: Pathway and Network Analysis

**Figure 3:**
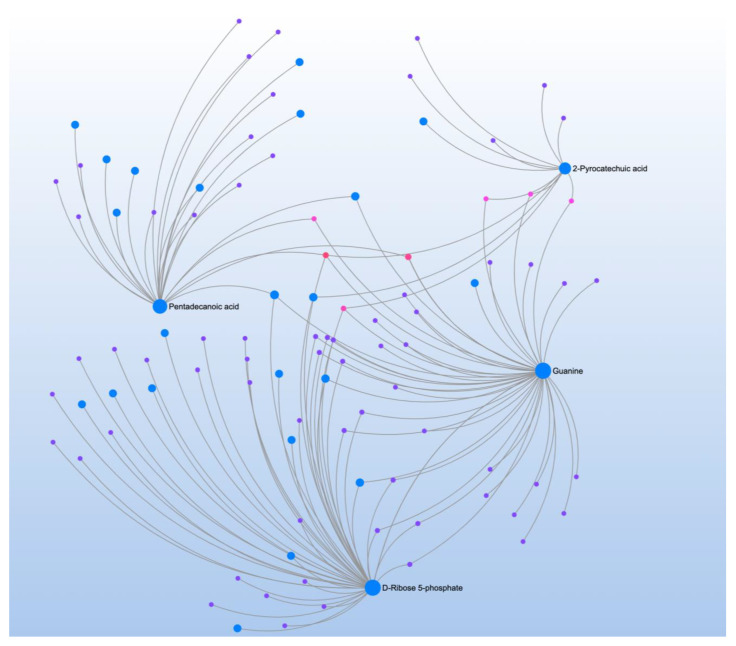
Metabolite-metabolite interaction network for significant metabolites that correlate to sleep problems. Size of the node indicates importance in the network. The interaction analysis confirmed the role of D-ribose 5-phosphate, an essential product of the pentose phosphate pathway that can be used to synthesize guanosine triphosphate and adenosine triphosphate.

CSHQ correlated with 25 metabolites that mapped to three pathways (Table 5). Metabolite-metabolite interaction analysis confirmed the role of D-ribose 5-phosphate, an essential product of the pentose phosphate pathway that can be used to synthesize guanosine triphosphate (GTP) and ATP (Figure 3). The analysis confirmed the role of guanine, which is the nucleobase of GTP and found two central metabolites: 2-pyrocatechuic acid (benzoic acid metabolite) and pentadecanoic acid (straight-chain saturated fatty acid).

**Figure 4:**
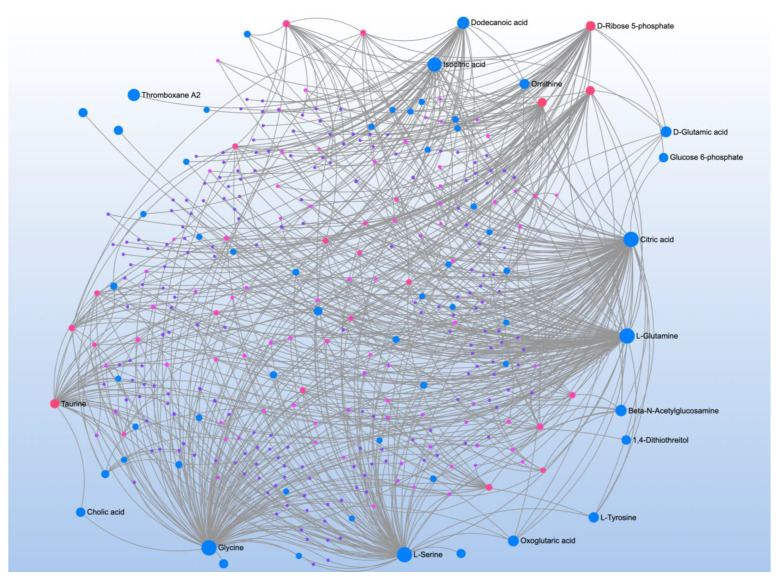
Metabolite-metabolite interaction network for significant metabolites that correlate to neurodevelopment. Size of the node indicates importance in the network. The interaction analysis highlights with citric acid cycle intermediates, citric acid and isocitric acid, as well as glucogenic amino acids, such as L-serine, glycine, and L-glutamine, highlighting the role of energy metabolism and the mitochondria specifically.

The VABS was correlated with 28 metabolites that mapped to four pathways (Table 6). Metabolite-metabolite interaction analysis highlighted the role of D-ribose 5-phosphate and other glucogenic amino acids, such as L-serine, glycine, and L-glutamine (Figure 4). The metabolite-metabolite interaction analysis also highlighted two citric acid cycle (CAC) organic acids, citric acid and isocitric acid, confirming the important role of the mitochondria.

**Figure 5:**
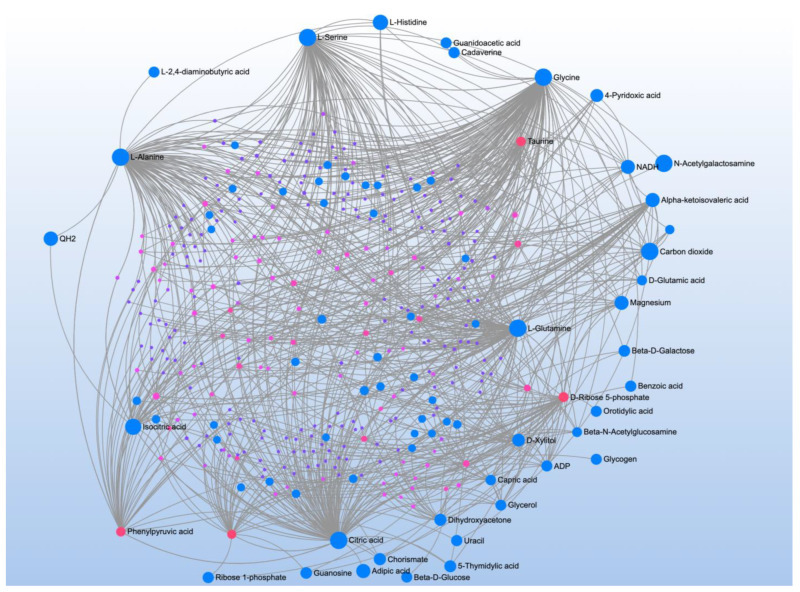
Metabolite-metabolite interaction network for significant metabolites that correlate to autism symptoms. Size of the node indicates importance in the network. The interaction analysis highlights with citric acid cycle metabolites such citric acid and carbon dioxide as well as glucogenic amino acids, such as L-serine, glycine, and L-glutamine, highlighting the role of energy metabolism and the mitochondria specifically.

The SRS correlated with 30 metabolites that mapped to 7 significant pathways (Table 7). Metabolite-metabolite interaction analysis confirmed the central role of D-ribose 5-phosphate and highlighted the metabolic and signaling roles of various CAC metabolites, such as citrate and isocitrate (Figure 5). This analysis also underlined the major role of the neurotransmitter, glycine; the glucogenic amino acids, L-alanine, L-glutamine, and L-serine; and the ketoacids, alpha-ketoisovaleric acid and phenylpyruvate. These metabolites were matched to various significant pathways such as glyoxylate and dicarboxylate metabolism; aminoacyl-tRNA biosynthesis; alanine, aspartate and glutamate metabolism; and glycine, serine and threonine metabolism.

**Figure 6:**
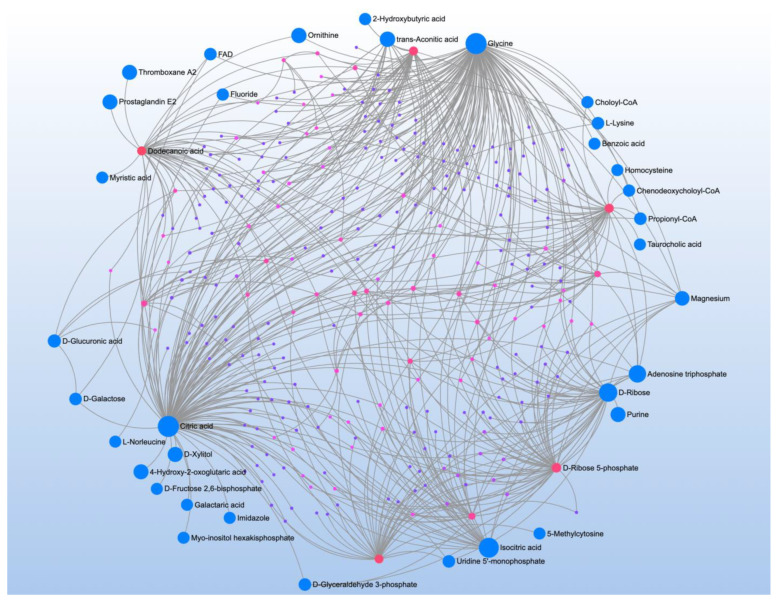
Metabolite-metabolite interaction network for significant metabolites that correlate to Language ability. Size of the node indicates importance in the network. The interaction analysis reveals the importance of both energy and purine metabolism.

The CL correlated with 8 metabolites that mapped to two significant pathways (Table 8). Metabolite-metabolite interaction analysis highlighted the roles of ATP and ribose 5-phosphate (Figure 6). The roles of the CAC organic acids citric and isocitric acid were again highlighted. Additionally, dodecanoic acid (medium chain fatty acid) and glycine (neurotransmitter) were confirmed as central metabolites.

**Figure 7:**
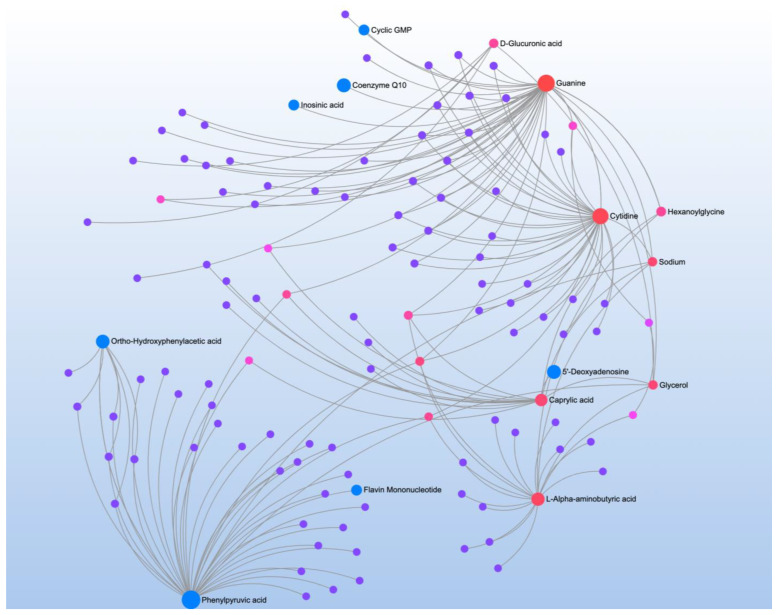
Metabolite-metabolite interaction network for significant metabolites that correlate with aberrant behavior. Size of the node indicates importance in the network. The interaction analysis highlights the importance of nucleotide metabolism as well as phenylalanine metabolism.

The ABC was correlate with 14 metabolites that mapped to two significant pathways: phenylalanine metabolism and AAA biosynthesis (Table 9). Metabolite-metabolite interaction analysis highlighted the central role of guanine (a component of GTP), cytidine (a component of RNA), and caprylic acid (a medium-chain fatty acid) (Figure 7). 

### 3.3. Correlations with Targeted Metabolites: Pathway and Network Analysis

**Figure 8:**
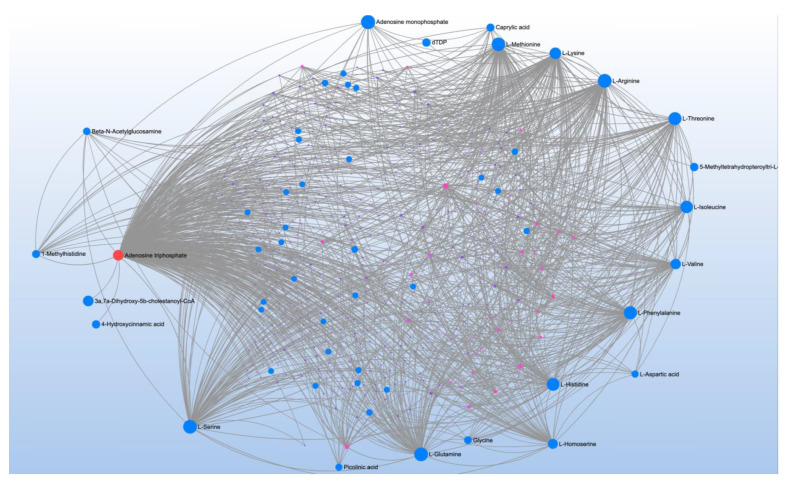
Metabolite-metabolite interaction network for significant metabolites that correlate methylation potential (S-adenosylmethionine to S-adenosylhomocysteine ratio). Size of the node indicates importance in the network. The interaction analysis highlights the importance of energy metabolism including glucogenic and ketogenic amino acids.

Methylation potential correlated with 28 metabolites with mapping to 6 pathways (Table 10). Metabolite-metabolite interaction analysis highlighted ATP; the glucogenic amino acids, L-methionine, L-arginine, L-threonine, L-isoleucine, L-valine, L-phenylalanine, L-histidine, L-glycine, L-glutamine and L-serine; and the ketogenic amino acid, L-lysine (Figure 8). The most significant pathway that these amino acids matched to was aminoacyl-tRNA biosynthesis.

**Figure 9:**
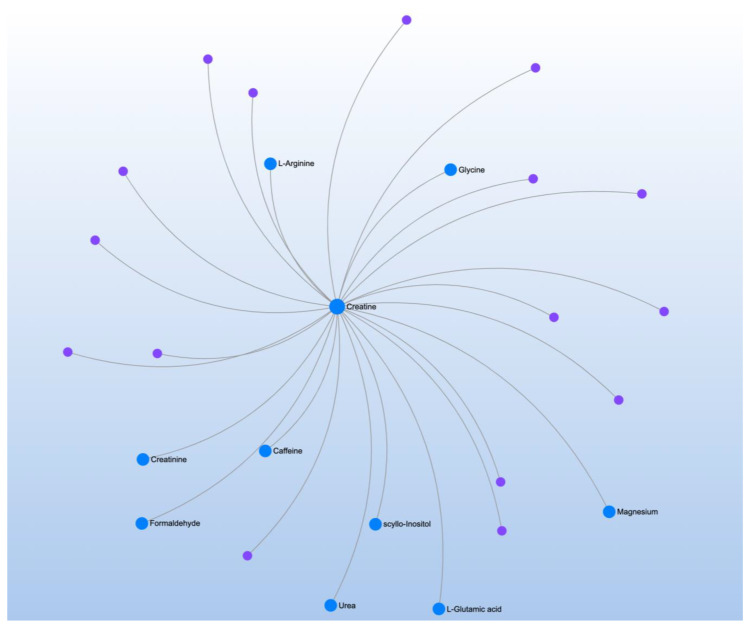
Metabolite-metabolite interaction network for significant metabolites that correlate to total glutathione redox ratio. Size of the node indicates importance in the network. The interaction analysis highlights the importance of creatine, an essential organic compound for maintaining and storing energy.

tGSH/GSSG correlated with two significant metabolites and mapped to two significant pathways (Table 11). The only central metabolite discriminated in the metabolite-metabolite interaction analysis was creatine, which is an essential organic compound for maintaining and storing energy (Figure 9).

**Figure 10:**
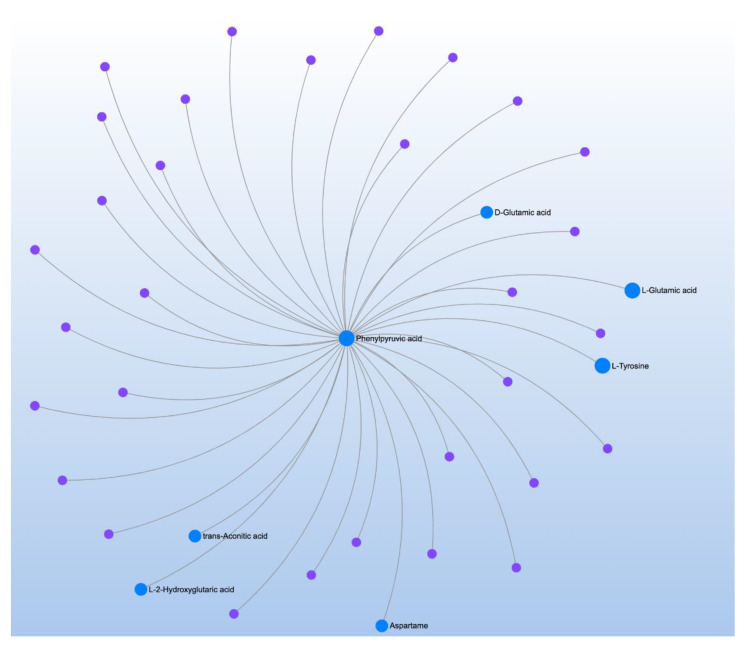
Metabolite-metabolite interaction network for significant metabolites that correlate to free glutathione redox. Size of the node indicates importance in the network. The interaction analysis highlights the importance of phenylpyruvic acid, implicating phenylalanine metabolism.

fGSH/GSSG correlated with two significant metabolites and two significant pathways, interesting the same pathways associated with aberrant behavior (Table 12). The ketoacid phenylpyruvic acid was highlighted in the metabolite-metabolite interaction analysis (Figure 10).

**Figure 11:**
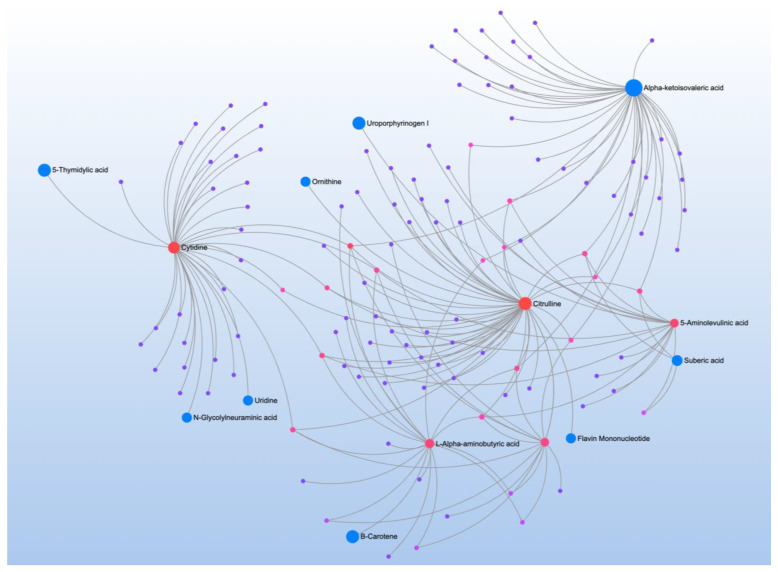
Metabolite-metabolite interaction network for significant metabolites that correlate to intracellular reduced glutathione redox ratio. Size of the node indicates importance in the network. The interaction analysis demonstrated various pathways including nucleotide, urea cycle and branched chain amino acid metabolism.

iGSH-GSSG was correlated with 7 metabolites and mapped to one pathway (Table 13). Metabolite-metabolite interaction analysis identified alpha-ketoisovaleric acid (keto acid), cytidine (a component of RNA), citrulline (non-essential amino acid), 5-aminolevulinic acid (responsible for heme production in mammals), and L-alpha-aminobutyric acid (product of the catabolism of methionine, threonine, and serine) as central metabolites (Figure 11).

**Figure 12:**
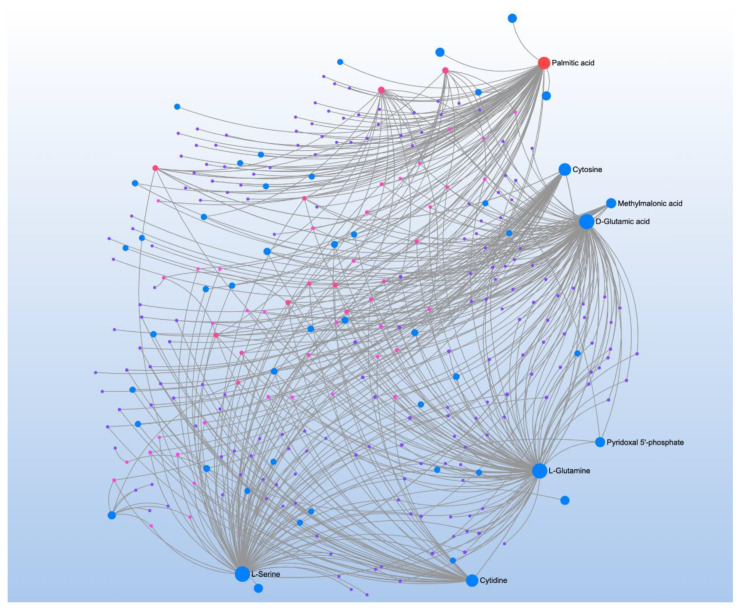
Metabolite-metabolite interaction network for significant metabolites that correlate to 3-nitrotyrosine, a biomarker of oxidative protein damage. Size of the node indicates importance in the network. The interaction analysis highlights the importance of amino acid neurotransmitter metabolism, particularly glutamine metabolism.

3-NT correlated with 16 metabolites and four pathways (Table 14). Metabolite-metabolite interaction analysis found palmitic acid, the most common saturated fatty acid in the body; cytosine, a nucleobase in DNA and RNA; and cytidine, a component of RNA as central metabolites (Figure 12). Additionally, this analysis identified three amino acids as central metabolites, D-glutamic acid, L-Glutamine, and L-serine. These amino acids were found to be matched in three of the four total significant pathways.

**Figure 13:**
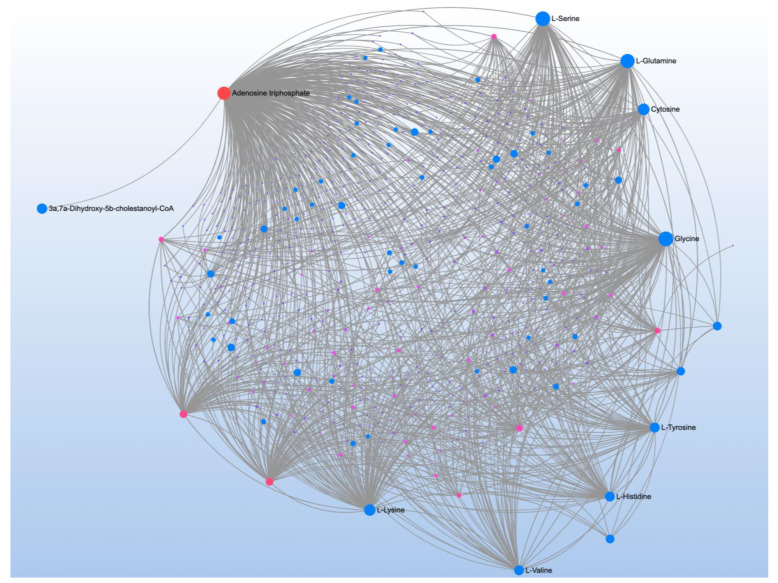
Metabolite-metabolite interaction network for significant metabolites that correlate to 3-chlorotyrosine, a biomarker of inflammation. Size of the node indicates importance in the network. The interaction analysis highlights the importance of the energy metabolism, particularly amino acid which feed into the citric acid cycle.

3-CT correlated with 31 metabolites and mapped to three pathways (Table 15). Metabolite-metabolite interaction analysis identified ATP and cytosine as well as the amino acids: L-serine, L-glutamine, glycine, L-tyrosine, L-histidine, L-valine, and L-lysine (Figure 13). These metabolites hint at mitochondrial function as they all have metabolic pathways associated with the mitochondria, though L-histidine is converted into pyruvate, the starting point of the CAC. Additionally, the majority of the amino acid central metabolites are matched to the aminoacyl-tRNA biosynthesis.

**Figure 14:**
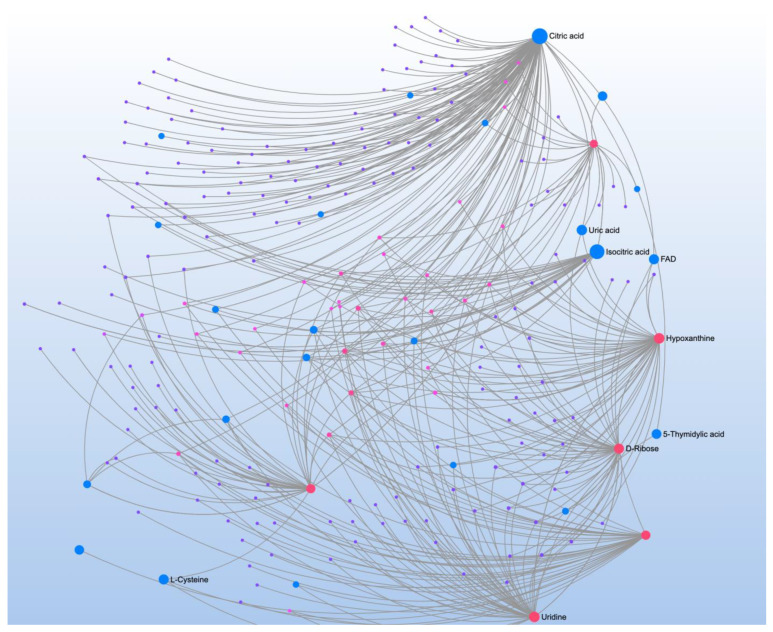
Metabolite-metabolite interaction network for significant metabolites that correlate to adenosine triphosphate-linked respiration, an index of mitochondrial energy production. Size of the node indicates importance in the network. The interaction analysis highlights the importance of citric acid cycle intermediates.

ALR correlated with 14 metabolites and two pathways (Table 16). Metabolite-metabolite interaction analysis highlighted the role of citric acid and isocitric acid, two CAC intermediates (Figure 14).

**Figure 15:**
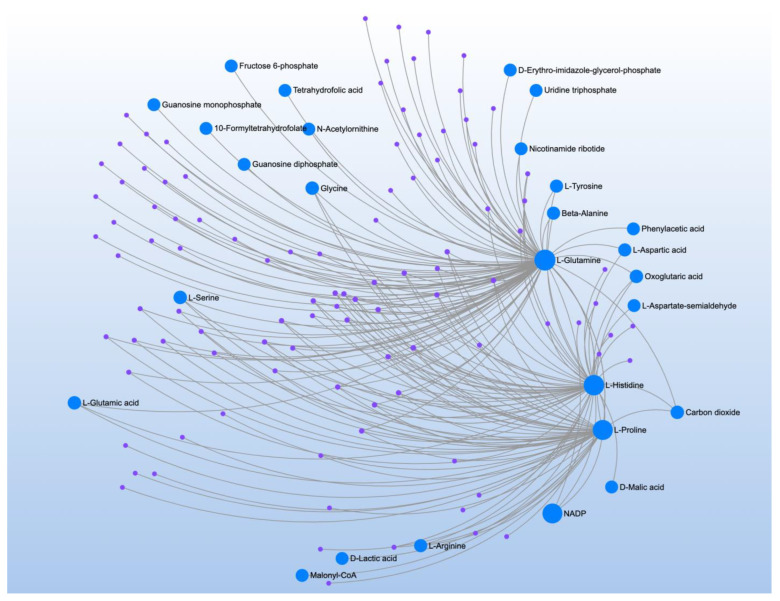
Metabolite-metabolite interaction network for significant metabolites that correlate to proton-leak respiration, a marker of regulation of oxidative stress at the mitochondrial inner membrane. Size of the node indicates importance in the network. The interaction analysis highlights the importance of glucogenic amino acids.

PLR correlated with 7 metabolites and mapped to three pathways (Table 17). Three glucogenic amino acids, L-glutamine, L-histidine, and L-proline, were identified in the metabolite-metabolite interaction analysis (Figure 15).

## 4. Discussion

This study used large-scale targeted LC-MS/MS metabolomic analysis to examine the metabolic profiles in blood from 57 children with ASD (29 with NDR) and compared these metabolic profiles to TD controls of similar age and gender. Furthermore, we examined the associations between metabolic measurements and behavioral and neurodevelopmental measurements and targeted metabolic biomarkers known to be abnormal in children with ASD. 

### 4.1. Summary of Results 

As a group, those with ASD were compared to TD controls, and the NDR subgroups were compared to each other and to TD controls, separately. Overall, 23 metabolites were found to be significantly different between ASD participants and TD controls representing significant differences in three pathways, histidine and GSH metabolism, as well as AAA biosynthesis. Nicotinamide metabolism differentiated those with ASD with NDR from those without NDR. The AAAs differentiated TD controls from individuals with ASD without NDR. Interesting, a different metabolic signature was found for those with NDR when compared to TD controls. Those with a history of NDR demonstrate disruption of aminoacyl-tRNA biosynthesis and histidine metabolism, as well as metabolism of glyoxylate and dicarboxylate (energy metabolism) and glycine, serine and threonine (energy and purine metabolism). Thus, these results suggest that subsets of children with ASD have distinct metabolism profiles specifically dependent on their developmental profile (NDR vs. no NDR). 

To better understand the heterogeneity of children with ASD, variations in behavior and neurodevelopment were correlated with changes in metabolism. Abnormalities in sleep were directly associated with disruption is energy metabolism, specifically CAC metabolism as well as the pentose phosphate pathway, an important source of nicotinamide adenine dinucleotide phosphate (NADPH). Overall neurodevelopment, as measured by the VABS, found that an association with energy and purine metabolism as well as aminoacyl-tRNA biosynthesis. Language was associated not only with a primary disruption in energy pathways but also in glycine, an amino acid neurotransmitter. ASD symptoms, as measured by the SRS, was found to be associated with energy, BCAA and glutamate metabolism, as well as aminoacyl-tRNA biosynthesis. Interestingly, disruptive behavior was primarily associated with AAA metabolism, implicating monoamine neurotransmitters. Thus, from this analysis, neurodevelopment metrics seems to be associated with energy metabolism, but ASD and aberrant behavior appears to be more related to amino acid metabolism and monoamine neurotransmitters.

Correlations between targeted measures of ASD metabolism and metabolomics were also considered. A measure of methylation potential, the SAM/SAH ratio, was found to be related to a wide range of metabolic processes, including very strongly related to aminoacyl-tRNA biosynthesis and related to BCAA, histidine and nicotinamide metabolism, as well as disruption in amino acids related to methylation such as methionine. Interestingly, the total GSH redox ratio was related to glycine, serine, threonine, arginine and proline metabolism through its relation to disruption of creatine while the free GSH redox ratio was associated with changed in AAA metabolism. The intracellular GSH redox ratio was related to BCAAs. 

Oxidative stress, as measured by 3-NT, was found to be related to glutamine, glycine, serine and threonine metabolism, as well as CAC and fatty acid energy metabolism. Metabolic-metabolic interaction analysis highlighted the importance of vitamin B6 and B12 in this network as both pyridoxal-5-phosphate and methylmalonic acid were highlighted nodes in the network. Interestingly, two D-amino acids, including D-glutamic acid and D-glutamate, which are related to bacterial metabolism were associated with 3-NT. 3-CT, a marker of immune activation, was found to be related to a wide range of metabolic pathways, including a very strong relationship to aminoacyl-tRNA biosynthesis and also histidine, glycine, serine and threonine metabolism, as well as CAC energy metabolism. Metabolic-metabolic interaction analysis highlighted the involvement of ATP and L-glutamine in these pathways. As expected, ALR, a measure of the mitochondrial ATP production, was associated with CAC metabolites. Interestingly, PLR, a measure of the mitochondria’s handling of oxidative stress at the inner mitochondrial membrane, was found to be related to glutamine metabolism. 

### 4.2. Energy Metabolism 

Disruption in energy metabolism was demonstrated in several different comparative analyses of ASD and TD individuals and in the correlations with behavior, neurodevelopment, and targeted metabolic pathways. Energy pathways were found to differentiate individuals with ASD and NDR and be related to sleep, overall neurodevelopment, language, and ASD symptoms (SRS). Interestingly, methylation and GSH metabolism did not seem to be connected to energy yet measures of oxidative stress and immune activation were related. Not surprisingly, ALR, a measure of mitochondrial production of energy, was strongly linked to the CAC while proton linked respiration was linked to glutamate metabolism. This latter finding is interesting as glutamate is an alternative fuel for the mitochondrial that is understudied in mitochondrial research of developmental disorders.

The repeated association of energy metabolism with many aspects of ASD is not surprising given the high rate of mitochondrial dysfunction associated with ASD [10,11]. Furthermore, the link to neurodevelopment and language is not surprising as previous studies have linked developmental delays to abnormal CAC and energy pathway biomarkers in urine [54], plasma [55,56], and CSF [34]. Children with ASD and mitochondrial disease appear to have two developmental profiles, those with developmental delays, including motor delay, and those that manifest NDR [10]. Our current study has linked energy metabolism abnormalities to both subgroups as abnormalities in energy metabolism differentiated those with NDR from TD controls and correlated with several measures of neurodevelopment. 

Interestingly, pathways other than central CAC metabolites were also identified. For example, disruption in glyoxylate and dicarboxylate pathway was identified in several analyses. This is an interesting pathway as it provides a short cut between the proximal CAC (isocitrate) and distal CAC (succinate, malate), thereby bypassing α-ketoglutarate and succinyl-CoA. These two intermediates that are bypassed are important in ASD physiology as propionic acid, an important short chain fatty acid associated with ASD symptoms [13,57], enters the CAC through succinyl-CoA, and α-ketoglutarate is closely metabolically connected to glutamate, an important neurotransmitter that is well known to be dysregulated in ASD.

Dysregulation of glycine, serine and threonine pathways is connected to the CAC through serine that is directly metabolized into pyruvate. Disruption in serine metabolism has been linked to ASD in the past [58], and glycine is an important amino acid neurotransmitter in the cortex and is a building block (precursor) to GSH. It is important to note that mitochondria are an important regulator of amino acids, as the urea cycle, the nitrogen disposal system, is partially located in the mitochondrial matrix, and BCAA (leucine, isoleucine, and valine) are key metabolic intermediates of mitochondrial metabolism. 

### 4.3. Amino Acid Neurotransmitter Metabolism

Several pathways were associated with changes in the AAA (Phenylalanine, tyrosine, and tryptophan) biosynthesis. Examining the metabolite changes demonstrates that this was driven by phenylpyruvate, a product which accumulates when phenylalanine breakdown is reduced, usually when hydroxylase activity is inhibited. This enzyme and other the hydroxylases required to metabolize AAA precursor into monoamine neurotransmitters are dependent on tetrahydrobiopterin as a cofactor, and low tetrahydrobiopterin levels are known to be associated with ASD [29]. Treatment trials of ASD children with tetrahydrobiopterin improve ASD behaviors [29] and the redox state [59]. Parallel to this, our analysis found that abnormalities in AAA biosynthesis were related to aberrant behavior and redox regulation as well as differentiated children with ASD and NDR from NT controls. 

L-Glutamine was found to be related to measure of neurodevelopment (VABS), ASD symptoms (SRS), as well as markers of methylation, oxidative, inflammation, and mitochondrial control of oxidative stress (PLR). Glutamine is a central amino acid which connects neurotransmission, redox metabolism, and mitochondrial function. Glutamine is the precursor/product of both glutamate, the main cortical excitatory neurotransmitter, and gamma-aminobutyric acid (GABA), the main cortical inhibitory neurotransmitter. As excitatory/inhibitory balance is known to be disrupted in ASD through these two neurotransmitters [60], glutamate balance is critical in ASD. Balance of these neurotransmitter is essential as balanced glutamate transmission is essential for learning and has been linked to many psychiatric symptoms such as repetitive behaviors which, in part, define ASD. Thus, the connection of glutamine to neurodevelopment and ASD symptoms found in this study is consistent with its physiological role. 

Glutamate is an essential component of GSH which is the body’s major antioxidant, consistent with the findings in this study that it is associated with measures of oxidative damage and inflammation. One of the products of the methylation cycle is homocysteine which can be metabolized into cysteine. Cysteine is one of the major three components of GSH, along with glutamate and glycine. As such, glutamate is connected to methylation and control of oxidative stress through the production of GSH.

Interestingly, in several of the analysis, glutamine was also related to markers of mitochondrial function. As mentioned above, glutamine is a mitochondrial substrate for producing energy which enters the CAC through α-ketoglutarate. The repeated findings of an activation of the glyoxylate and dicarboxylate pathway which bypasses this portion of the CAC suggests an increase in glutamine is entering the CAC. Thus, this suggests a close association of the neurotransmitter regulation and mitochondrial function in ASD.

Glycine is an inhibitory neurotransmitter with anti-inflammatory, cytoprotective, and immune modulating properties [61]. In this study, glycine was associated with the NDR subtype of ASD and measures of sleep and ASD symptoms, neurodevelopment and GSH metabolism, and a marker of inflammation. Interesting, mitochondrial metabolism was also associated with the same factors (except for 3CT). Glycine is connected to mitochondrial metabolism in several ways. Glycine combines with Succinyl-CoA, a CAC intermediate, to produce 5-aminolevulinate, a precursor used for heme synthesis which is important for the function of cytochromes used in the electron transport chain complexes. Glycine is also involved in mitochondrial one-carbon metabolism and is essential to produce purines. Glycine is also interconverted into CO_2_ and ammonium. Thus, aside from being a neurotransmitter, glycine is involved in several other critical pathways that could influence brain function and development. 

### 4.4. Branched Chain Amino Acid Metabolism

BCAA biosynthesis was found to correlate with ASD symptoms, methylation potential and intracellular GSH ratio, although with low pathway impact scores. It is estimated that approximately 17% of children with ASD have relative reduced concentrations of BCAAs [22]. This finding is supposedly similar to Branched Chain Ketoacid Dehydrogenase Kinase deficiency, an inborn error of metabolism, which is characterized by ASD, epilepsy, and intellectual disability [62]. However, unlike studies on mice in which the animal responded to BCAA supplementation, such a simple treatment has not been shown to be successfully therapeutic in humans. Branch chain amino acids have diverse physiological roles including modulating glucose and fatty acid metabolism as well as regulating important molecular pathways and promoting protein synthesis, and they are connected to mitochondrial function by feeding into the CAC through succinyl-CoA [63]. Thus, BCAAs appear to may have an interesting role in ASD, although their integral role in ASD physiology needs to be better elucidated. 

### 4.5. Nicotinamide Metabolism

NDR was associated with changes in nicotinamide metabolism. Previous studies have found that urine nicotinamide metabolites may be a biomarker for ASD [32] and may indicate increased nicotinamide degradation [33]. NADP and its redox couple (NADPH) are cofactors for many important metabolic pathways [64]. NADP is important in CAC enzymes, pyruvate metabolism, mitochondrial proton-translocation, folate metabolism and glutamate deamination. NADPH is essential for fatty acid synthesis, steroidogenesis, drug metabolism and heme degradation [64,65], ubiquinol production [66] and thioredoxin reductases function [67]. Interestingly, mitochondrial NADP biosynthesis has been linked to mitochondrial redox regulation [68] and has been linked to a mitochondrial disease phenotype [68]. NAD precursors have been shown to have therapeutic effect on mitochondrial function [69]. Given that NDR has been linked to a unique type of mitochondrial dysfunction [46], it is possible that NAD has a role in the dysregulation of mitochondrial function in NDR. 

### 4.6. Aminoacyl-tRNA Biosynthesis

Variations in aminoacyl-tRNA biosynthesis were found in participants with ASD and a history of NDR and were related to measures of neurodevelopment, ASD symptoms, methylation potential, and immune activation. Aminoacyl-tRNAs, tRNAs bonded to their cognate amino acid, serve as key substrates responsible for translating the genetic code into proteins. Synthesis of these tRNAs directly relies on the aminoacyl-tRNA synthase [70]. Thus, aminoacyl-tRNA biosynthesis is a key component for producing key proteins for cellular functions. Other aspects of tRNA function have also been linked to ASD. For example, ASD-associated regulatory SNPs have been found to impact aminoacyl-tRNA biosynthesis in the fetal cortex, impacting additional pathways such as ribosome biogenesis [71]. Thus, disruption of tRNA function may underly the biological mechanisms of ASD in some individuals. 

### 4.7. Histidine

Disruptions in histidine metabolism differentiated TD controls from those with ASD, particularly those with ASD and a history of NDR. Methylation potential and 3-CT, a marker of immune activation, were also strongly related to the histidine metabolism. The strong correlation between the 3-CT measurement and histidine metabolism may be explained by how histamine, an integral of the immune system, can be easily synthesized by decarboxylation of histidine [72]. Various studies have reported changes in the immune systems of children with ASD [73]. Histidine is a precursor of carnosine, a dipeptide that contains beta alanine and histidine, in the human brain, where it acts as a buffer and antioxidant [72]. Carnosine has been shown to regulate various biological pathways connected to intellectual disabilities [74] and has been shown to be therapeutic for in controlled studies [75,76,77]. In addition, multiple researchers have looked at Histidinemia, or elevated histidine concentration in the blood, and have shown a relationship between histidinemia and ASD [78,79] and language delay [80,81].

### 4.8. Common Pathways

Interestingly, the data from this study converged on several pathways. One interesting aspect of the many of the pathways and metabolites is that many of the pathways were all connected to various intermediates within the CAC. As energy metabolism, particularly with respect to the mitochondria and CAC, is repeatedly identified in many of the pathway analysis, it is very possible that the majority of the non-mitochondrial pathways found to be disrupted could be caused by or the cause of mitochondrial dysfunction through disruption of the flux of metabolites in the CAC. This could explain the high prevalence of mitochondrial dysfunction without the findings of mutation is mitochondrial genes. Specifically, disruption in function of these non-mitochondrial pathways could be the cause of secondary mitochondrial dysfunction in many individuals with ASD. A better understanding of these connections between mitochondrial and non-mitochondrial pathways could lead to improved treatments for individuals with ASD.

### 4.9. Limiations

This study has several limitations, particularly the limited sample size, providing only a limited ability to understand the heterogeneity of ASD. In order to account for this, the variation in behavior and other specific metabolic disruptions associated with ASD were correlated with metabolites measured. To investigate the relationship between metabolic signatures and mitochondrial function parameters derived from whole fresh intact cellular based respirometry were examined. However, in the future, additional markers could be examined. The study participants were derived from a natural history study of individuals with ASD with much of the data obtained retrospectively. Thus, future cohorts derived from prospective early life histories would increase the strength of the study.

## 5. Conclusions

This study examined the metabolic profiles of ASD in relation to several behavior, neurodevelopmental and targeted metabolic biomarkers in an effort to uncover a metabolic signature related to ASD. We used both pathway analysis and metabolomic-metabolomic interactions to examine metabolites. Energy and amino acid pathways were commonly disrupted with many of the amino acids being closely linked to energy production. 

This study had several limitations including selection of samples from general samples of ASD which were not preselected to have a specific range of severity and a limited number of controls. Further studies should use larger sample sizes to better characterize the patients and a greater number of controls. Simultaneous collection of urine and stool samples may be very useful to better understand how systematic and microbiome changes can influence metabolism.

## Figures and Tables

**Table 1:** Metabolites and their pathways which are different between autism spectrum disorder and control participants.

Significant Metabolites		Significant Pathways		
Metabolite	Fold ∆	Pathway	Matched Metabolites	Impact
Cytidine ***	7.17	Histidine metabolism *	L-Histidine *, 1-Methylhistamine **	0.31
Taurine **	4.52	Phenylalanine, tyrosine and tryptophan biosynthesis	Phenylpyruvate	0.05
5-Aminolevulinic acid **	0.22			
Dodecanoic acid **	0.26	Glutathione metabolism	Glycine **, Pyroglutamic acid *	0.10
Glycine **	3.77			
1-Methylhistamine **	3.72			
Adenosine triphosphate **	3.65			
2,3,4,5-Tetrahydroxypentanoic acid **	3.63			
4-Hydroxyproline **	0.28			
Dihydroxyacetone **	3.27			
4-Pyridoxic acid **	3.26			
Phenylpyruvic acid **	0.31			
2-Aminoisobutyric acid **	3.22			
Xylitol **	3.21			
Isobutyric acid *	3.20			
Capric acid *	0.31			
D-Leucic acid *	0.32			
L-Histidine *	3.17			
DL-Acetylcarnitine *	3.10			
Ribitol *	3.05			
L-3-Phenyllactic acid *	0.33			
Pyroglutamic acid *	2.83			
4-Hydroxybenzaldehyde *	0.37			

* *p* ≤ 0.05, ** *p* ≤ 0.01; *** *p* ≤ 0.001. Metabolites in green are higher in the ASD sample while those in red are reduced in the ASD sample.

**Table 2:** Metabolites and their pathways which are different between participants with and without neurodevelopmental regression.

Significant Metabolites		Significant Pathways		
Metabolite	Fold ∆	Pathway	Matched Metabolites	Impact
2-Pyrocatechuic acid *	0.26	Nicotinate/nicotinamide metabolism *	Niacinamide *	0.19
Niacinamide *	3.92			
Acetamide *	3.74			

* *p* ≤ 0.05. Metabolites in green are higher in the neurodevelopmental regression groupe while those in red are reduced in those without neurodevelopmental regression.

**Table 3:** Metabolites and their pathways which are different between autism spectrum disorder without neurodevelopmental regression as compared to control participants.

Significant Metabolites		Significant Pathways		
Metabolite	Fold ∆	Pathway	Matched Metabolites	Impact
5-Aminolevulinic acid ***	0.15	Phenylalanine, tyrosine and tryptophan metabolism *	Phenylpyruvic acid *	0.00
Cytidine ***	6.12			
4-Hydroxyproline **	0.17			
4-Pyridoxic acid **	4.50			
2,3,4,5-Tetrahydroxypentanoic acid **	4.31			
D-Leucic acid *	0.26			
L-3-Phenyllactic acid *	0.27			
Ribitol *	3.66			
Isobutyric acid *	3.49			
Xylitol *	3.48			
4-Hydroxybenzaldehyde *	0.29			
Taurine *	3.27			
1-Methylhistamine *	3.23			
2-Aminoisobutyric acid *	3.22			
4-Hydroxybutyric acid *	0.31			
Capric acid *	0.32			
Phenylpyruvic acid *	0.33			
Dodecanoic acid *	0.33			

* *p* ≤ 0.05, ** *p* ≤ 0.01; *** *p* ≤ 0.001.

**Table 4:** Metabolites and their pathways which are different between autism spectrum disorder with neurodevelopmental regression and control participants.

Significant Metabolites		Significant Pathways		
Metabolite	Fold ∆	Pathway	Matched Metabolites	Impact
Cytidine ***	9.66	Aminoacyl-tRNA biosynthesis ***	L-Asparagine *, L-Histidine *, Glycine **, L-Serine **	0.17
Taurine ***	7.54			
Adenosine triphosphate ***	6.86	Histidine metabolism **	L-Histidine*, 1-Methylhistamine *	0.31
Glycine **	6.49	Glyoxylate and dicarboxylate metabolism *	L-Serine **, Glycine **	0.15
Dodecanoic acid **	0.20	Glycine, serine and threonine metabolism *	L-Serine **, Glycine **	0.46
D,L-Acetyl-carnitine **	4.84			
Dihydroxyacetone **	4.76			
Acetamide **	4.69			
L-Histidine **	4.60			
Hydroxykynurenine **	4.54			
L-Serine **	4.49			
1-Methylhistamine *	4.16			
L-Asparagine *	3.94			
trans-Aconitic acid *	0.28			
2,3,4,5-Tetrahydroxypentanoic acid *	3.49			
Capric acid *	0.30			
Heptadecanoic acid *	3.33			
2-Aminoisobutyric acid *	3.31			
Isobutyric acid *	3.27			

* *p ≤* 0.05, ** *p ≤* 0.01; *** *p ≤* 0.001.

**Table 5:** Metabolites and their pathways which correlate with sleep disruption.

Significant Metabolites		Significant Pathways		
Metabolite	r	Pathway	Matched Metabolites	Impact
Isocitric acid ***	0.37	Citrate cycle (TCA cycle) **	Isocitrate ***, Citrate **, Fumarate *	0.17
Glucuronic acid ***	0.36	Glyoxylate and dicarboxylate metabolism *	Citrate **, Glycine *, Isocitrate ***	0.14
Citric acid **	0.35	Pentose phosphate pathway *	D-Ribose 5-phosphate **, D-Ribose *	0.17
Phenylpyruvic acid **	0.33			
3S-methyl-2-oxo-pentanoic acid **	0.32			
Phenylbutazone **	−0.32			
trans-Aconitic acid **	0.31			
2-Hydroxybutyric acid **	−0.30			
Cytidine **	−0.29			
Amiloride **	−0.29			
D-Ribose 5-phosphate **	−0.28			
4-Hydroxyproline *	0.27			
Glycine *	−0.26			
Citraconic acid *	0.26			
D-Ribose *	0.26			
Ketoleucine *	0.26			
1-Methylhistamine *	−0.25			
Dodecanoic acid *	0.25			
Pregnenolone sulfate *	−0.25			
5-Aminolevulinic acid *	0.24			
2-Methylglutaric acid *	−0.24			
Taurine *	−0.24			
L-3-Phenyllactic acid *	0.24			
Fumaric acid *	0.23			
N-Acetylglutamine *	−0.22			
Dihydroxyacetone *	−0.22			

* *p ≤* 0.05, ** *p ≤* 0.01; *** *p ≤* 0.001.

**Table 6:** Metabolites and their pathways which correlate with neurodevelopment.

Significant Metabolites		Significant Pathways		
Metabolite	r	Pathway	Matched Metabolites	Impact
Phenylpyruvic acid **	0.35	Glyoxylate and dicarboxylate metabolism ***	Citrate *, L-Serine **, Glycine **, Isocitrate *, L-Glutamine *	0.18
Cytidine **	−0.35			
Glycine **	−0.33	Glycine, serine and threonine metabolism *	L-Serine **, Glycine **, 5-Aminolevulinate	0.46
Taurine **	−0.33			
Phenylbutazone **	−0.32	Citrate cycle (TCA Cycle) *	Isocitrate *, Citrate *	0.14
Amiloride **	−0.31	Aminoacyl-tRNA biosynthesis *	L-Glutamine *, Glycine **, L-Serine **	0.17
Glucuronic acid **	0.31			
L-Serine **	−0.30			
3S-methyl-2-oxo-pentanoic acid **	0.30			
DL-Acetylcarnitine **	−0.30			
1-Methylhistamine **	−0.30			
L-3-Phenyllactic acid **	0.29			
Dihydroxyacetone *	−0.29			
L-Glutamine *	−0.28			
Dodecanoic acid *	0.27			
Citraconic acid *	0.27			
Isocitric acid *	0.27			
4-Pyridoxic acid *	−0.26			
Capric acid *	0.26			
Ketoleucine *	0.26			
Pregnenolone sulfate *	−0.26			
5-Aminolevulinic acid *	0.25			
Isovaleric acid *	−0.25			
2-Aminoisobutyric acid *	−0.24			
Citric acid *	0.24			
4-Hydroxyproline *	0.24			
D-Leucic acid *	0.23			
D-Ribose 5-phosphate *	−0.22			

* *p ≤* 0.05, ** *p ≤* 0.01; *** *p ≤* 0.001.

**Table 7:** Metabolites and their pathways which correlate with autism symptoms.

Significant Metabolites		Significant Pathways		
Metabolite	r	Pathway	Matched Metabolites	Impact
Phenylpyruvic acid ***	−0.44	Glyoxylate and dicarboxylate Metabolism ***	Citrate *, L-Serine **, Glycine *, Isocitrate *, L-Glutamine *	0.18
Cytidine ***	0.42			
5-Aminolevulinic acid ***	−0.39	Valine, leucine and isoleucine Biosynthesis **	3-Methyl-2-oxobutanoic acid *, 4-Methyl-2-oxopentanoate ***	0
3S-methyl-2-oxo-pentanoic acid ***	−0.38			
4-Hydroxyproline **	−0.36	Aminoacyl-tRNA biosynthesis **	L-Glutamine *, Glycine *, L-Serine **, L-Alanine *	0.17
L-3-Phenyllactic acid **	−0.36			
Taurine **	0.35	Alanine, aspartate and glutamate Metabolism *	L-Alanine *, L-Glutamine *, Citrate *	0.11
Citraconic acid **	−0.35			
Ketoleucine **	−0.35	Glycine, serine and threonine Metabolism *	L-Serine **, Glycine *, 5-Aminolevulinate ***	0.46
1-Methylhistamine **	0.33			
D-Leucic acid **	−0.32	Pentose and glucuronate Interconversions *	Xylitol *, D-Glucuronate **	0.3
Glucuronic acid **	−0.32			
Amiloride **	0.30	Citrate cycle (TCA Cycle) *	Isocitrate *, Citrate *	0.14
L-Serine **	0.29			
Xylitol *	0.29			
Isocitric acid *	−0.28			
Glycine *	0.27			
DL-Acetylcarnitine *	0.25			
L-Glutamine *	0.25			
Dihydroxyacetone *	0.25			
trans-Aconitic acid *	−0.25			
Citric acid *	−0.25			
Capric acid *	−0.24			
L-Alanine *	−0.24			
2-Aminoisobutyric acid *	0.24			
Isobutyric acid *	0.23			
2,3,4,5-Tetrahydroxypentanoic acid *	0.23			
4-Pyridoxic acid *	0.23			
D-Ribose 5-phosphate *	0.23			
Alpha-ketoisovaleric acid *	−0.22			

* *p ≤* 0.05, ** *p ≤* 0.01; *** *p ≤* 0.001.

**Table 8:** Metabolites and their pathways which correlate with language ability.

Significant Metabolites		Significant Pathways		
Metabolite	r	Pathway	Matched Metabolites	Impact
Creatinine **	−0.40	Pentose and glucuronate interconversions	L-Arabitol *, Xylitol *	0.17
Ribitol *	0.33			
Galactitol *	0.32	Galactose metabolism	Galactitol *, D-Sorbitol *	0
Sorbitol *	0.31			
L-Arabitol *	0.30			
Xylitol *	0.30			
Mannitol *	0.30			
Phenylbutazone *	−0.30			

* *p ≤* 0.05, ** *p ≤* 0.01.

**Table 9:** Metabolites and their pathways which correlate with aberrant behavior.

Significant Metabolites		Significant Pathways		
Metabolite	r	Pathway	Matched Metabolites	Impact
L-3-Phenyllactic acid ***	−0.39	Phenylalanine metabolism **	Phenylpyruvate **, 2-Hydroxyphenylacetate *	0.26
Phenylpyruvic acid **	−0.35			
D-Leucic acid **	−0.34	Phenylalanine, tyrosine and tryptophan biosynthesis *	Phenylpyruvate **	0
Guanine *	−0.26			
3-Hexenedioic acid *	−0.25			
L-Arabinose *	0.25			
Ortho-Hydroxyphenylacetic acid *	−0.25			
Amiloride *	0.24			
Glucuronic acid *	−0.24			
3S-methyl-2-oxo-pentanoic acid *	−0.23			
Cytidine *	0.23			
L-Alpha-aminobutyric acid *	−0.23			
3-Hydroxyisovaleric acid *	−0.22			
Caprylic acid *	−0.22			

* *p ≤* 0.05, ** *p ≤* 0.01; *** *p ≤* 0.001.

**Table 10:** Metabolites and their pathways which correlate with methylation potential.

Significant Metabolites		Significant Pathways		
Metabolite	r	Pathway	Matched Metabolites	Impact
Methylguanidine ***	0.41	Aminoacyl-tRNA biosynthesis ***	L-Asparagine *, L-Histidine *, L-Phenylalanine **, L-Arginine **, L-Glutamine *, L-Serine *, L-Methionine **, L-Valine *, L-Lysine ***, L-Isoleucine **, L-Threonine ***, L-Proline **	0.17
L-Threonine ***	0.41			
L-Homoserine ***	0.41			
L-Lysine ***	0.36			
L-Isoleucine **	0.33			
L-Arginine **	0.31			
Caprylic acid **	0.29	Valine, leucine and isoleucine biosynthesis ***	L-Threonine ***, L-Isoleucine ***, L-Valine *	0
Adenosine triphosphate **	0.29			
L-Methionine **	0.29	Cysteine and methionine metabolism *	L-Serine *, L-Methionine **, (S)-2-Aminobutanoate*	0.17
Indolelactic acid **	0.29			
2-hydroxyglutaric acid **	0.28	Arginine biosynthesis *	L-Arginine ***, L-Glutamine *	0.08
L-Proline **	0.27	Nicotinate and nicotinamide metabolism *	Quinolinate *, Nicotinamide *	0.19
L-Phenylalanine **	0.27	Histidine metabolism *	L-Histidine *, N(pi)-Methyl-L-histidine *	0.22
L-Alloisoleucine **	0.27			
L-Kynurenine *	0.26			
L-Asparagine *	0.26			
L-Alpha-aminobutyric acid *	0.26			
1-Methylhistidine *	0.25			
5-Hydroxyindoleacetic acid *	0.25			
Quinolinic acid *	0.25			
L-Valine *	0.24			
L-Glutamine *	0.24			
L-Serine *	0.23			
L-Histidine *	0.22			
Capric acid *	0.22			
Niacinamide *	0.21			
L-3-Phenyllactic acid *	0.21			
Picolinic acid *	0.20			

* *p* ≤ 0.05, ** *p* ≤ 0.01; *** *p* ≤ 0.001.

**Table 11:** Metabolites and their pathways which correlate with the total glutathione redox ratio.

Significant Metabolites		Significant Pathways		
Metabolite	r	Pathway	Matched Metabolites	Impact
Creatine *	−0.26	Glycine, serine and threonine metabolism *	Creatine *	0
Nonadecanoic acid *	0.21	Arginine and proline metabolism *	Creatine *	0.01

* *p* ≤ 0.05.

**Table 12:** Metabolites and their pathways which correlate with the free glutathione redox ratio.

Significant Metabolites		Significant Pathways		
Metabolite	r	Pathway	Matched Metabolites	Impact
Nonadecanoic acid *	0.24	Phenylalanine, tyrosine and tryptophan biosynthesis **	Phenylpyruvate *	0
Phenylpyruvic acid *	0.22			
		Phenylalanine metabolism *	Phenylpyruvate *	0.26

* *p* ≤ 0.05, ** *p* ≤ 0.01.

**Table 13:** Metabolites and their pathways which correlate with the intracellular glutathione redox ratio.

Significant Metabolites		Significant Pathways		
Metabolite	r	Pathway	Matched Metabolites	Impact
Suberic acid **	0.31	Valine, leucine and isoleucine *	3-Methyl-2-oxobutanoic acid	0
Cytidine *	−0.26			
5-Aminolevulinic acid *	0.25			
L-Alpha-aminobutyric acid *	0.24			
Citrulline *	0.24			
4-Hydroxyproline *	0.23			
Alpha-ketoisovaleric acid *	−0.21			

* *p* ≤ 0.05, ** *p* ≤ 0.01.

**Table 14:** Metabolites and their pathways which correlate with oxidative damage.

Significant Metabolites		Significant Pathways		
Metabolite	r	Pathway	Matched Metabolites	Impact
Stearic acid ***	0.34	D-Glutamine and D-glutamate metabolism **	D-Glutamate **, L-Glutamine *	0.5
Palmitic acid **	0.30			
Isobutyric acid **	0.29	Glyoxylate and dicarboxylate metabolism *	L-Serine **, L-Glutamine *	0.04
Cytidine **	0.28	Glycine, serine and threonine metabolism *	L-Serine **, 5-Aminolevulinate *	0.22
D-Leucic acid **	−0.27	Biosynthesis of unsaturated fatty acids *	Palmitic acid **, Octadecanoic acid	0
L-Serine **	0.27			
D-Glutamic acid **	0.27			
4-Pyridoxic acid *	0.26			
5-Aminolevulinic acid *	−0.24			
L-Glutamine *	0.22			
4-Hydroxyproline *	−0.22			
Cytosine *	0.22			
Methylmalonic acid *	−0.22			
Acetamide *	0.21			
N-Acetylethanolamine *	0.21			
2-Methylglutaric acid *	−0.20			

* *p* ≤ 0.05, ** *p* ≤ 0.01; *** *p* ≤ 0.001.

**Table 15:** Metabolites and their pathways which correlate with a marker of inflammation.

Significant Metabolites		Significant Pathways		
Metabolite	r	Pathway	Matched Metabolites	Impact
L-Glutamine ***	0.47	Aminoacyl-tRNA biosynthesis***	L-Asparagine ***, L-Histidine ***, L-Glutamine ***, Glycine **, L-Serine ***, L-Valine *, L-Lysine *, L-Tyrosine **, L-Proline ***	0.17
L-Serine ***	0.42			
L-Proline ***	0.39			
L-Histidine ***	0.39			
DL-Acetylcarnitine ***	0.38	Glyoxylate and dicarboxylate metabolism*	L-Serine ***, Glycine **, L-Glutamine ***	0.15
Palmitic acid ***	0.37			
Acetamide ***	0.37	Glycine, serine and threonine metabolism*	L-Serine ***, Glycine **, 5-Aminolevulinate *	0.46
L-Asparagine ***	0.34			
Stearic acid **	0.33	Histidine metabolism*	L-Histidine ***, Imidazole-4-acetate *	0.22
D-Leucic acid **	−0.33			
Epinephrine **	0.32			
Taurine **	0.30			
6-Methyl-DL-Tryptophan **	0.29			
Cytidine **	0.28			
3-Hexenedioic acid **	−0.27			
5-Hydroxyindoleacetic acid **	0.27			
L-Tyrosine **	0.27			
Glycine **	0.27			
Adenosine triphosphate **	0.27			
Imidazoleacetic acid *	0.26			
Dihydroxyacetone *	0.26			
Isobutyric acid *	0.25			
Cytosine *	0.23			
L-Alloisoleucine *	0.23			
5-Aminolevulinic acid *	−0.23			
Methylmalonic acid *	−0.22			
Picolinic acid *	0.22			
2-hydroxyglutaric acid *	0.22			
L-Valine *	0.21			
L-Lysine *	0.21			
N-Acetylglutamine *	0.21			

* *p* ≤ 0.05, ** *p* ≤ 0.01; *** *p* ≤ 0.001.

**Table 16:** Metabolites and their pathways which correlate with adenosine triphosphate linked respiration.

Significant Metabolites		Significant Pathways		
Metabolite	r	Pathway	Matched Metabolites	Impact
Hypoxanthine ***	−0.34	Citrate cycle (TCA cycle) **	Isocitrate *, Citrate *	0.14
3-Hexenedioic acid **	0.31	Glyoxylate and dicarboxylate metabolism *	Citrate *, Isocitrate *	0.03
Pyroglutamic acid **	−0.27			
D-Ribose **	0.27			
Uridine **	−0.27			
L-Arabinose *	−0.26			
Citric acid *	0.25			
Isocitric acid *	0.24			
Glucuronic acid *	0.23			
Salicylic acid *	0.22			
2-Methylglutaric acid *	−0.22			
Creatine *	−0.21			
Nonadecanoic acid *	0.20			
N-Acetylglutamine *	−0.20			

* *p* ≤ 0.05, ** *p* ≤ 0.01; *** *p* ≤ 0.001.

**Table 17:** Metabolites and their pathways which correlate with proton-leak respiration.

Significant Metabolites		Significant Pathways		
Metabolite	r	Pathway	Matched Metabolites	Impact
L-Proline **	0.28	Aminoacyl-tRNA biosynthesis ***	L-Histidine *, L-Glutamine *, L-Proline **	0
Acetamide *	0.26			
3-Hydroxyisovaleric acid *	0.26	D-Glutamine and D-glutamate metabolism *	L-Glutamine *	0
L-Glutamine *	0.25			
Ribitol *	0.22	Nitrogen metabolism *	L-Glutamine *	0
L-Histidine *	0.21			
DL-Acetylcarnitine *	0.21			

* *p* ≤ 0.05, ** *p* ≤ 0.01; *** *p* ≤ 0.001.

## Data Availability

Data is available upon request.

## Supplementary Materials

The following supporting information can be downloaded at: https://www.mdpi.com/article/10.3390/jpm12101727/s1, Figure S1: Data Normalization for Individual Features; Table S1: Participant Characteristics.
