# Supplementary material for: Metabolomic Signatures of Autism Spectrum Disorder
Source: J Pers Med. 2022 Oct 17;12(10):1727. doi: 10.3390/jpm12101727 (PMC9604590; doi:10.3390/jpm12101727)
Supplement: Supplementary file 1 [file jpm-12-01727-s001.zip › Table S1.pdf]

| Table S1. Participant Characteristics | | | |
|---|---|---|---|
| Variable | Typically Developing | ASD without Regression | ASD with Regression |
| Number of Subjects | 37 | 28 | 29 |
| Age, mean (SD), years months | 8 y 2 m (4 y 4 m) | 8y 4m (3y 9m) | 8y 0m (3y 6m) |
| Females, N (%) | 17 (46%) | 14 (50%) | 4 (14%) |
| Regression, N (%) | | | |
| Single Regression | | | 19 (65%) |
| Age at 1st Regression | | | 22m (7m) |
| Loss of Language Skills | | | 23 (79%) |
| Loss of Social Skills | | | 24 (83%) |
| Loss of Gross Motor Skills | | | 8 (28%) |
| Loss of Fine Motor Skills | | | 7 (24%) |
| Associated Fever | | | 6 (21%) |
| Associated Illness | | | 10 (34%) |
| Associated Seizure | | | 3 (10%) |
| Associated Other | | | 10 (35%) |
| Associated Any Trigger | | | 18 (62%) |
| Neurodevelopmental and Behavior Score Mean (St Dev) | | | |
| Language Ability (Scaled Score) | 102 (12) | 74 (26) | 63 (24) |
| Vineland Adaptive Behavior Scale (Scaled Score) | | | |
| • Communication Subscale | 107 (14) | 69 (18) | 63 (20) |
| • Daily Living Skills Subscale | 105 (10) | 67 (15) | 64 (15) |
| • Social Subscale | 112 (11) | 68 (15) | 62 (14) |
| • Motor Subscale | 108 (13) | 78 (18) | 75 (14) |
| • Adaptive Behavioral Composite | 108 (11) | 66 (14) | 62 (15) |
| Social Responsiveness Scale (T-Score) | | | |
| • Awareness | 51 (11) | 73 (12) | 75 (14) |
| • Cognitive | 49 (10) | 82 (11) | 78 (10) |
| • Communication | 47 (8) | 80 (13) | 81 (11) |
| • Motivation | 49 (10) | 72 (13) | 78 (13) |
| • Mannerisms | 47 (11) | 80 (18) | 80 (12) |
| • Total | 47 (10) | 83 (12) | 83 (11) |
| Aberrant Behavior Checklist (Raw Score) | | | |
| • Irritability | 3 (4) | 17 (10) | 13 (9) |
| • Lethargy / Social Withdrawal | 1 (2) | 14 (9) | 13 (8) |
| • Stereotyped Movements | 0 (1) | 8 (6) | 6 (5) |
| • Hyperactivity | 4 (7) | 24 12) | 20 (11) |
| • Inappropriate Speech | 0 (1) | 4 (3) | 3 (3) |
| Comorbid Conditions (Parent Report), N (%)* | | | |
| Neurologic | 3/26 (12%) | 19/27 (70%) | 17/26 (65%) |
| Allergic | 7/26 (27%) | 15/27 (56%) | 13/26 (50%) |
| Psychiatric | 2/26 (8%) | 20/27 (74%) | 13/26 (50%) |
| Gastrointestinal | 6/26 (23%) | 15/27 (56%) | 11/26 (42%) |
| Immune | 1/26 (4%) | 7/27 (26%) | 7/26 (27%) |
| Growth | 3/26 (12%) | 10/27 (37%) | 8/26 (31%) |
| Endocrine | 0/26 (0%) | 1/27 (4%) | 3/26 (12%) |
| Cardiovascular | 0/26 (0%) | 2/27 (7%) | 2/26 (8%) |
| Comorbid Conditions (Medical Records), N (%)* | | | |
| Food Allergies/Intolerances | 5/36 (14%) | 19/25 (76%) | 19/26 (73%) |
| Epilepsy | 0/37 (0%) | 6/28 (21%) | 13/29 (45%) |
| Chronic Constipation | 4/34 (12%) | 13/23 (57%) | 11/28 (39%) |
| Fatigue/Exercise Intolerance | 2/37 (5%) | 6/28 (21%) | 11/29 (38%) |
| Recurrent Infections (AAAAI Criteria) | 4/36 (11%) | 4/20 (20%) | 8/28 (29%) |
| Gross Motor Delay | 1/37 (3%) | 13/28 (46%) | 16/29 (55%) |
| Hypogammaglobinemia | 0/6 (0%) | 2/21 (10%) | 1/25 (4%) |
| Failure to Thrive | 0/37 (0%) | 2/28 (7%) | 5/29 (17%) |
| Genetic Syndrome | 0/37 (0%) | 2/28 (7%) | 0/29 (0%) |
| Treatments, N (%) | | | |
| Gastrointestinal Medications | 6 (16%) | 12 (43%) | 13 (45%) |
| Melatonin | 0 (0%) | 9 (32%) | 7 (24%) |
| Allergy/Asthma Medications | 3 (8%) | 8 (29%) | 18 (40%) |
| Mineral Supplements | 0 (0%) | 6 (21%) | 5 (17%) |
| Antiepileptic Medications | 0 (0%) | 3 (11%) | 8 (28%) |
| Antimicrobial Medications | 0 (0%) | 3 (11%) | 3 (11%) |
| Other Psychotropic Medications | 0 (0%) | 3 (11%) | 4 (14%) |

| | | | |
|---|---|---|---|
| Immunomodulatory Medications | 1 (3%) | 2 (7%) | 6 (21%) |
| Stimulant | 0 (0%) | 8 (29%) | 4 (14%) |
| Alpha-adrenergic agonists | 0 (0%) | 3 (11%) | 6 (21%) |
| Selective Serotonin Reuptake Inhibitors | 0 (0%) | 3 (11%) | 2 (7%) |
| Hormone Supplementation | 0 (0%) | 1 (4%) | 1 (3%) |
| Thyroid Supplementation | 0 (0%) | 1 (4%) | 1 (3%) |
| Beta Blocker | 0 (0%) | 2 (7%) | 2 (7%) |
| Dietary Formula | 0 (0%) | 1 (4%) | 1 (3%) |

*Some families did not answer every survey question and some information could not be abstracted from medical chart so not every patient is represented in these percentages.
