# Supplementary figures and images for: Metabolomic Signatures of Autism Spectrum Disorder
Source: J Pers Med. 2022 Oct 17;12(10):1727. doi: 10.3390/jpm12101727 (PMC9604590; doi:10.3390/jpm12101727)

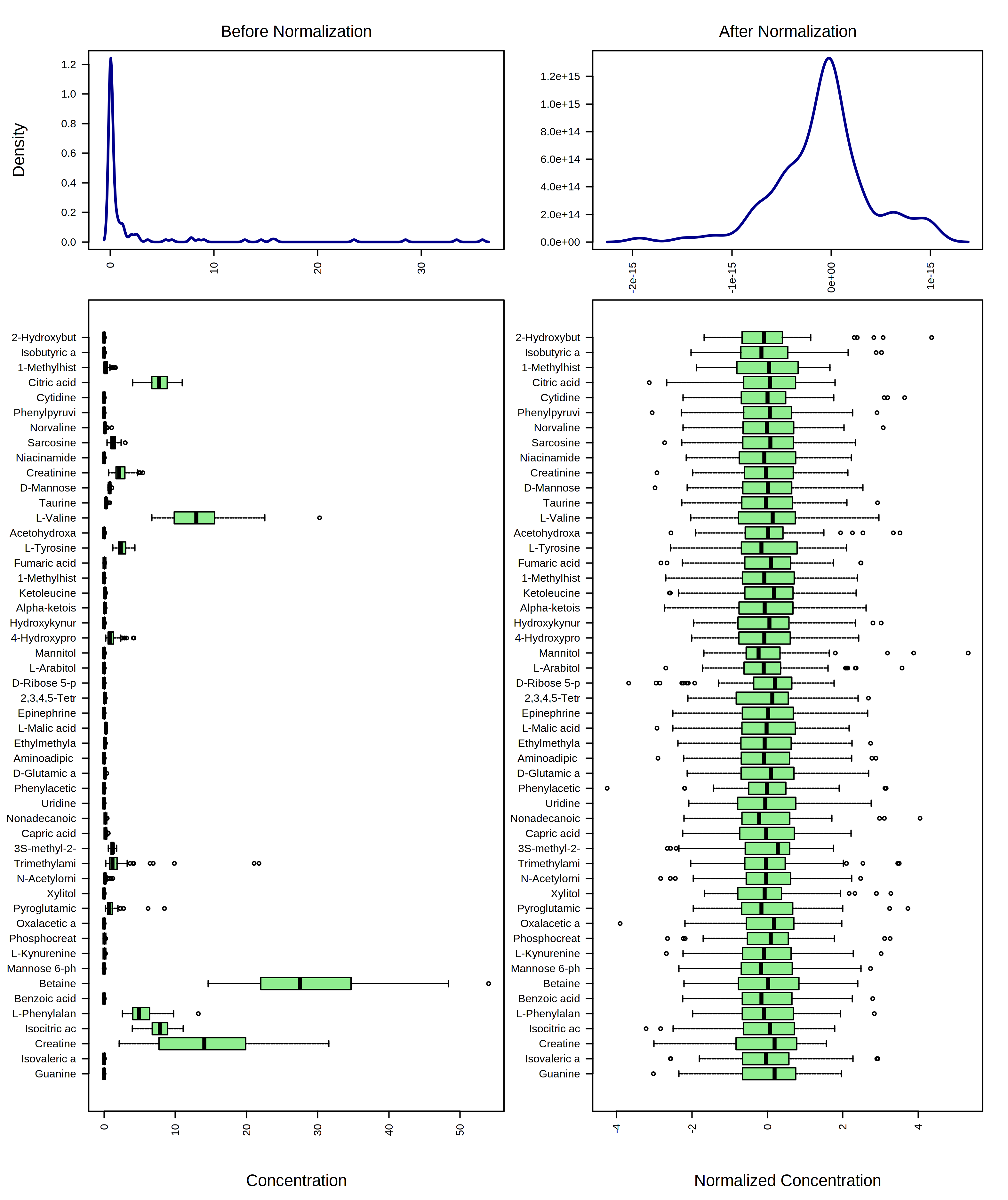

Supplement: Supplementary file 1 [file jpm-12-01727-s001.zip › Figure S1 normalization.png]
